# Systematic Review of Randomized Clinical Trials on Safety and Efficacy of Pharmacological and Nonpharmacological Treatments for Retinitis Pigmentosa

**DOI:** 10.1155/2015/737053

**Published:** 2015-08-03

**Authors:** Marta Sacchetti, Flavio Mantelli, Daniela Merlo, Alessandro Lambiase

**Affiliations:** ^1^Cornea and Ocular Surface Unit, San Raffaele Hospital IRCCS, Via Olgettina, No. 60, 20132 Milan, Italy; ^2^Department of Biology, College of Science and Technology, Temple University, 1900 N. 12th Street, Philadelphia, PA 19122, USA; ^3^Department of Cell Biology and Neuroscience, Istituto Superiore di Sanità, Viale Regina Elena 299, 00161 Rome, Italy; ^4^Department of Sense Organs, Sapienza University, Viale del Policlinico 155, 00186 Rome, Italy

## Abstract

*Aims.* Several treatments have been proposed to slow down progression of Retinitis pigmentosa (RP), a hereditary retinal degenerative condition leading to severe visual impairment. The aim of this study is to systematically review data from randomized clinical trials (RCTs) evaluating safety and efficacy of medical interventions for the treatment of RP.* Methods.* Randomized clinical trials on medical treatments for syndromic and nonsyndromic RP published up to December 2014 were included in the review. Visual acuity, visual field, electroretinogram, and adverse events were used as outcome measures.* Results.* The 19 RCTs included in this systematic review included trials on hyperbaric oxygen delivery, topical brimonidine tartrate, vitamins, docosahexaenoic acid, gangliosides, lutein, oral nilvadipine, ciliary neurotrophic factor, and valproic acid. All treatments proved safe but did not show significant benefit on visual function. Long term supplementation with vitamin A showed a significantly slower decline rate in electroretinogram amplitude.* Conclusions.* Although all medical treatments for RP appear safe, evidence emerging from RCTs is limited since they do not present comparable results suitable for quantitative statistical analysis. The limited number of RCTs, the poor clinical results, and the heterogeneity among studies negatively influence the strength of recommendations for the long term management of RP patients.

## 1. Introduction

Retinitis pigmentosa (RP) comprises a group of inherited progressive retinal dystrophies, characterized by rod and cone photoreceptor degeneration and progressive loss of peripheral and central vision. Retinitis pigmentosa is often diagnosed in children and young adults and, as the disease progresses and more photoreceptors degenerate, patients experience centripetal visual loss leading to legal and functional blindness [1–3].

Due to the progressive nature of the disease, there is great interest in the development of therapeutic interventions that may halt the evolution of the disease or restore the lost visual function. Currently, the therapeutic approach is restricted to slowing down the degenerative process, treating the ocular complications such as cataract and macular edema, and helping patients to cope with the social and psychological impact of blindness [1].

Nonpharmacological interventions are based on strategies of light protection as evidences indicate that some genetic types of pigmentary retinopathies are partly light-dependent [4]. Hyperbaric oxygen therapy has been also proposed for RP patients in order to promote photoreceptors survival [5, 6].

Several medical treatments have been proposed to slow down disease progression. Specifically, the trophic and antioxidant effects of vitamins have been evaluated in RP patients in order to demonstrate a protective action on photoreceptors [1, 7, 8]. Other nutritional supplementations, including docosahexaenoic acid (DHA), an omega 3 fatty acid found in high concentration in oil fish, lutein, and gangliosides are cited as a potential therapeutic modality that can help in preserving the visual function of patients with RP. Among them, DHA is considered important for photoreceptor function because membranes containing rhodopsin and cone opsins in photoreceptor cells have very high concentrations of this fatty acid, while the protective effect of lutein supplementation has been demonstrated in age-related macular degeneration [9, 10].

Other pharmacological treatments have been proposed for RP in small clinical studies, including oral valproic acid, oral nilvadipine, and beta-carotene [11–13]. Lastly, topical brimonidine tartrate 0.2% treatment and intravitreal delivery of ciliary neurotrophic factor (CNTF) were proposed for their neuroprotective effects observed in animal studies [14, 15].

The objective of this study was to systematically review scientific evidence currently available in the literature, in order to assess the effects of medical interventions for the treatment of patients with RP. All the randomized clinical trials for the evaluation of any medical treatments for RP published up to December 2014 were included in the review.

## 2. Materials and Methods

### 2.1. Literature Search

Six observers, divided in three groups of two, independently performed a literature search of all publication years up to December 2014. The articles were identified through a computerized search for clinical trials in the Cochrane Controlled Trial Register (CENTRAL/CCTR) (which contains the Cochrane Eyes and vision group trials register) on the Cochrane Library, Medline, and Embase. The search strategy was used to identify randomized clinical trials, as recommended by the Cochrane collaboration.

The following search strategy was used:

(a) Publication type was clinical trial.
(b) Keywords/search terms for disease were as follows: pigmentosa and retiniti^*∗*^, explode retinitis pigmentosa/all subheadings, RP/all subheadings, pigment^*∗*^ and retin^*∗*^, and explode pigmentary retinopathy/all subheadings.
(c) Keywords/search terms for medications were as follows: vit^*∗*^, explode vitamin A and/or vitamin E/all subheadings, retinol, retinyl palmitate, tocopherol, tocotrienol, lutein, carotenoid, omega 3/all subheadings, fatty acid/all subheadings, docosahexaenoic acid, DHA, cervonic acid, eicosapentaenoic acid, EPA, alpha-linolenic acid, ALA, alpha-2 agonist, alpha-2 adrenergic, brimonidine, clonidine, apraclonidine, ganglioside, valproic acid, nilvadipine, hyperbar^*∗*^, explode hyperbaric/all subheadings, neurotrophin, and neurotrophic factor.

In addition, linked references in all relevant articles were searched. The search resulted in a total of 389 abstracts.

### 2.2. Inclusion and Exclusion Criteria

#### 2.2.1. Type of Studies

Articles potentially eligible for inclusion in this systematic review were randomized clinical trials on medical therapy for RP with at least 4 weeks of follow-up published up to December 2014, written in English, French, German, Italian, Portuguese, or Spanish.

#### 2.2.2. Type of Participants

All participants who have been diagnosed with RP were included with no restrictions of age, gender, ethnicity, or use of adjunctive therapy. Trials evaluating patients with ocular comorbidities or complications that are known to influence visual function were excluded.

#### 2.2.3. Type of Interventions

Studies that specifically investigated the experimental medical interventions for RP such as vitamin A, vitamin E, omega 3 fatty acids (DHA), lutein, alpha-2 agonists, gangliosides, nilvadipine, valproic acid, CNTF, hyperbaric oxygen delivery (HBO), and light protection were included.

#### 2.2.4. Types of Comparisons

Any medical cointervention was considered.

Articles were excluded if they did not satisfy one or more inclusion criteria or if they were irretrievable after performing all available search strategies, including request to authors and editors.

**Figure 1:**
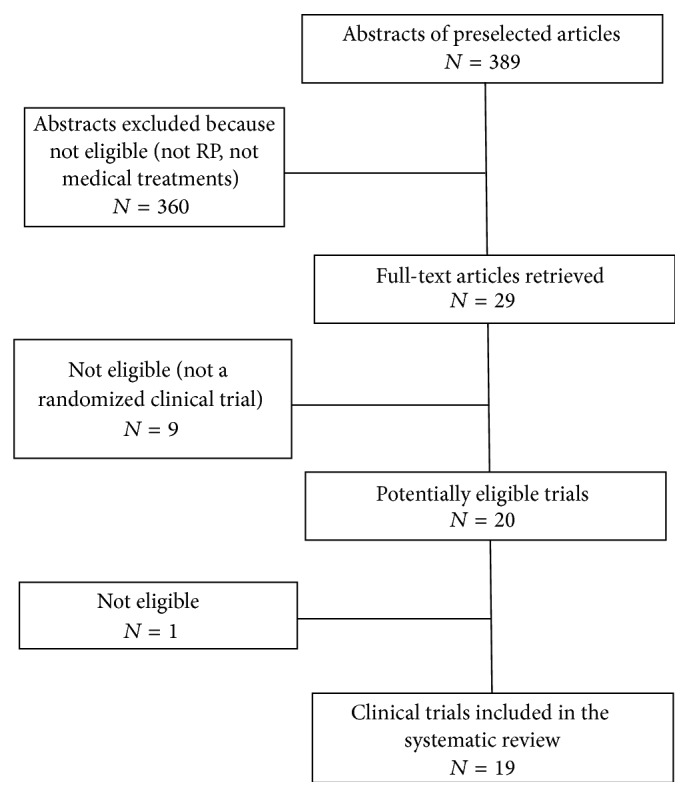
Decision tree of randomized clinical trials' selection for inclusion in the systematic review and meta-analysis.

The articles' eligibility was initially determined by evaluating the titles, abstracts, and MeSH (medical subject headings). Four observers divided into two groups of two examined all the retrieved 389 abstracts to consider their eligibility. After matching the decisions of the two groups, 360 abstracts were immediately excluded because they were either not randomized, not on medical treatment for RP, or related to different kinds of ocular disease. The remaining 29 complete articles were obtained and printed to identify whether they were suitable for inclusion in the revision and distributed to four researchers randomly divided into two groups of two each. The observers were blinded to the names of the authors and institutions, the name of the journals, the sources of funding, and the sponsors of the studies. The observers of each group were also blinded to the decisions of the other group and trial selection was matched between them. Nine trials were excluded because they did not match one or more inclusion criteria and one was excluded because it was not eligible [16–25]. All the remaining 19 RCTs were included in the systematic review [5, 6, 8–11, 13–15, 17, 26–34] (Figure 1).

### 2.3. Outcome Measures

Outcome measures included in this review are changes in visual field (Goldmann perimeter and Humprey visual field analyzer), best corrected visual acuity (BCVA), electroretinogram (ERG) amplitude, contrast sensitivity, dark adaptation, and treatment-related adverse events.

### 2.4. Assessment of Risk of Bias in Included Studies

*Validity Assessment.* Two authors independently assessed the included studies for sources of systematic bias according to the guidelines in section 6 of the Cochrane handbook for systematic reviews of interventions. Specifically, the following criteria were considered.

**Figure 2:**
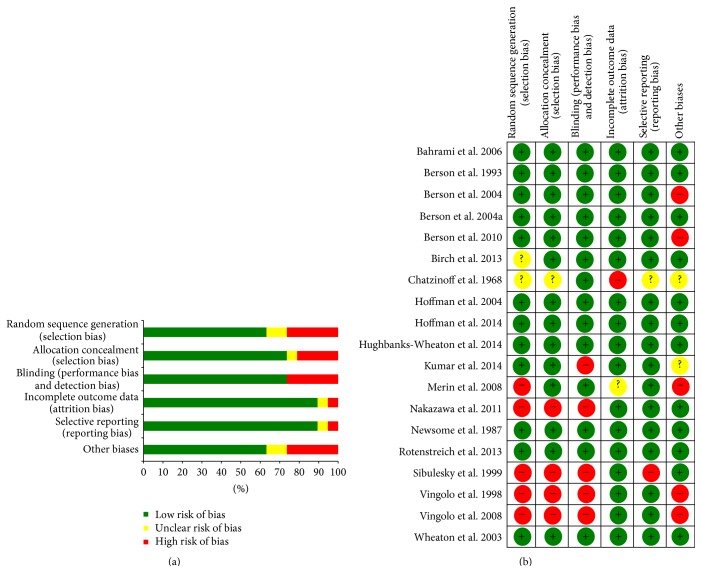
Risk of bias graph (a) showing authors' judgements about each risk of bias item presented as percentages across all included studies. Risk of bias summary (b) reviewing authors' judgements about each risk of bias item for each included study.

All studies included in the review were randomised controlled trials. The main quality attributes were scored as “low risk,” “high risk,” and “unclear risk” for the following areas: (i) whether or not the randomisation is properly concealed (random sequence generation and allocation concealment), (ii) whether or not the participants are masked (blinding), (iii) whether or not the outcome assessment is masked (incomplete outcome data), and (iv) for selective reporting bias. Other biases include the presence of commercial support, potential source of bias related to the specific study design used, or we are not sure whether an important risk of bias existed (Figure 2). 

*Data Synthesis.* We were unable to conduct meta-analyses on RP treatments because of the clinical heterogeneity observed between studies. Different interventions, different time points for outcome measures, and different instruments and methods of outcome evaluation meant that a summary effect was not estimated.

### 2.5. Statistical Analysis

RevMan5 software was used to analyse the data. We tested the heterogeneity between studies using the Chi-square test, with significant heterogeneity (*p* < 0.05) precluding meta-analysis.

## 3. Results

The 19 studies included in the systematic review dated from 1968 to 2014, all but one of these published in ophthalmic journals. Two trials were performed in Europe, 4 in Asia, and 13 in America. All were randomized clinical trials. Sixteen studies were double-masked or single- (investigator-) masked and three were unmasked (Table 1). RCTs included in this systematic review lasted from 4 months to 10 years with a mean duration of approximately three and a half years.

A total of 1774 patients with RP were enrolled in these studies. Demographic characteristics are summarized in Table 2. The 19 studies included in this systematic review evaluated the following interventions in RP patients: two on HBO [5, 6], one on topical brimonidine tartrate [14], five on vitamins supplementation [8, 26–28, 33], 4 on DHA supplementation [29–31, 34], 2 trials on lutein [9, 10], one on gangliosides' supplementation [32], one on nilvadipine [13], one on beta-carotene [11], one on CNTF [15], and one on oral valproic acid [12]. Results of the RCTs included in this review are summarized in Table 3.

While in some trials randomization appeared to have been executed properly, that is, an unpredictable sequence of treatment allocation was concealed adequately from people recruiting participants into the trial, the following biases have arisen in the trials included in this review (Figure 2):

- Sequence generation was not specified or performed in seven studies [5, 6, 13–15, 28, 33].
- Allocation concealment was not specified or performed in five trials [5, 6, 13, 28, 33].
- Five studies were unmasked [5, 6, 12, 13, 33].

### 3.1. Effects of Interventions on the Progression of RP

#### 3.1.1. Vitamin A Supplementation

Vitamin A supplementation did not show significant benefit on visual acuity, Goldman visual field, and dark adaptometry as compared to placebo in a first study by Chatzinoff et al. that evaluated 89 patients with RP for 3 years [28]. Later studies comparing vitamin A (15000 IU/day) and E (400 UI/day) versus placebo (traces amount of both vitamins) in a population of 601 patients with RP followed for 4 to 6 years reported that the groups receiving vitamin A showed a significantly slower rate of decline of ERG amplitude, which was not observed in patients receiving only traces amount of vitamins (placebo). The groups receiving vitamin A were 32% less likely to have a decline in ERG amplitude from baseline in a given year. Data from this study also showed that patients receiving vitamin E (400 UI/day) showed a greater loss of retinal functions than placebo groups [33, 35].

#### 3.1.2. Docosahexaenoic Acid (DHA) Supplementation

In a 4-year RCT by Hoffman et al., DHA (400 mg/die) treatment was not effective in improving visual acuity, visual field, ERG, and dark adaptometry when compared to placebo. In the same study population, Wheaton et al. reported that four-year DHA supplementation was associated with an acceptable safety profile [30, 34]. In a recent study, Hoffman et al. further evaluated the effects of oral DHA (30 mg/kg/day) supplementation versus placebo in a 4-year study in 78 patients with X-linked RP [29]. The loss rate of cone, rod, or maximal ERG function was not different between groups. In the same population, Hughbanks-Wheaton et al. confirmed the safety profile of long term DHA supplementation in the same population, with no differences in adverse events rate, antioxidant activity, platelet aggregation, or plasma lipoprotein levels between groups [29, 31].

An additional study from Berson et al. in 2004, in 221 patients with RP, showed that combined supplementation with vitamin A (15000 UI/day) and DHA (1200 mg/day) over a 4-year period did not slow the course of RP when compared to placebo in terms of visual field, visual acuity, and 30 Hz ERG changes [27]. A subsequent subgroup analysis performed by the same authors showed that, in RP patients not taking vitamin A therapy before entering the study, addition of DHA 1200 mg/d slowed the decline of visual field and ERG [26]. Specifically, the mean annual rate of decline of visual field was not significantly different between the placebo + vitamin A and DHA + vitamin A groups (30.26 ± 3.92 dB/year versus 39.41 ± 3.76 dB/year, *p* = 0.09), while patients not taking vitamin A prior to entry to the study showed significant differences between the placebo + vitamin A and DHA + vitamin A groups (52.5 ± 5.99 dB/year versus 30.7 ± 6.48 dB/year, *p* = 0.002). Similarly, the percentage of decline per year of 30 Hz ERG amplitude was not significantly different for those taking vitamin A prior to entry (9.23% in placebo + A and 10.57% in DHA + A groups), while patients not taking vitamin A prior to entry in the study showed a percentage of decline of 8.05% in the placebo + vitamin A group and of 12.99% in the DHA + vitamin A group, which was significant when comparing rates of decline in both groups of treatment (*p* = 0.02) [26].

#### 3.1.3. Lutein Supplementation

Two RCTs evaluated the effects of lutein supplementation in patients with RP when compared to placebo [9, 10]. Bahrami et al. demonstrated a positive effect of lutein supplementation on preserving visual filed in a 6-month study on 34 patients with RP. Specifically, lutein supplementation showed that the mean retinal area of the central visual field, evaluated by Goldman perimetry, was 0.018 log higher (*p* = 0.038) when compared to placebo [9]. A later study from Berson et al. evaluated 225 patients with RP treated with lutein (12 mg/day) or placebo in a 4-year study. All patients were given 15000 UI/day of vitamin A. This study showed no significant difference in visual acuity, ERG amplitude, and the rate of decline between the lutein plus vitamin A and control plus vitamin A groups for the HFA 30-2 program. For the HFA60-4 program, a decrease in mean rate of sensitivity loss was observed in the lutein plus vitamin A group (*p* = 0.05) [9, 10].

#### 3.1.4. Gangliosides Supplementation

One RCT by Newsome et al. demonstrated no significant effects of gangliosides administration when compared to placebo on the progression of RP evaluated by visual field and ERG in a 4-month study in 32 patients [32].

#### 3.1.5. Beta-Carotene Acid Supplementation

Oral administration of 9-cis *β*-carotene-rich alga* Dunaliella bardawil* (300 mg/day) was compared to placebo in a 3-month crossover study in 29 patients with RP. This study showed a significant improvement of maximal dark-adapted ERG b-wave amplitude responses when compared to placebo (+8.4 *µ*V versus −5.9 *µ*V, resp., *p* = 0.001) with 34.5% of patients showing an increase of more than 10 *µ*V for both eyes in the* Dunaliella* group. Light-adapted single-flash b-wave amplitudes were also significantly improved in* Dunaliella* group as compared to placebo (+17.8 *µ*V versus −3 *µ*V, resp., *p* = 0.01) while no significant changes were observed in visual acuity and visual field assessment [11].

#### 3.1.6. Oral Valproic Acid

One open randomized clinical trial by Kumar et al. evaluated the effects of 1 year of oral administration of valproic acid in 30 patients with typical RP. In this study, only evaluators were masked and 30 patients were randomized to valproic acid or no treatment. Visual acuity improved in the treatment group when compared to baseline and a statistically significant difference with controls was observed after 1 year (1.3 versus 1.83 LogMar, resp.; *p* ≤ 0.01). Multifocal ERG also showed a significant improvement in the valproic acid group (*p* ≤ 0.01) [12].

#### 3.1.7. Brimonidine 0.2% Eye Drops

Topical treatment with brimonidine tartrate 0.2% (Alphagan; Allergan, Irvine, CA) was administered to 17 patients with RP for 24/36 months. At the end of the study there were no significant benefits in visual field, visual acuity, and contrast sensitivity when compared to artificial tears [14].

#### 3.1.8. Ciliary Neurotrophic Factor Intraocular Implants

Encapsulated cell-ciliary neurotrophic factor intraocular implants were applied to one randomly selected eye of 133 patients included in two clinical trials on early-stage (CNTF4) and late-stage (CNTF3) RP. Implants were retained for 12 or 24 months, with 42 patients receiving low dose (5 ng/day) implants and 91 patients receiving high dose implants (20 ng/day). At the end of the study no significant differences in BCDVA and ERG amplitude were observed, and the high dose implants induced a significant worsening in Humphrey visual field sensitivity (CNTF3: −98.4 ± 165.3 high dose versus −14 ± 101.5 sham, *p* = 0.001; CNTF4: −164.3 ± 114.6 high dose versus −67.1 ± 104.2 sham, *p* < 0.001) [15].

#### 3.1.9. Oral Nilvadipine

A small nonmasked prospective study by Nakazawa et al. showed that oral nilvadipine (4 mg/daily) administration in 33 patients for 30–66 months with RP induced a slower progression of the visual field deterioration when compared to a control group receiving tocopherol nicotinate 300 mg/day or helenien 15 mg/day or no treatments (mean regression coefficient of the MD slope = −0.49 ± 0.17 dB/year versus −0.89 ± 0.16 dB/year, resp.; *p* = 0.042) [13].

#### 3.1.10. Hyperbaric Oxygen Delivery

The effects of HBO therapy were evaluated in two studies by Vingolo et al. The first study reported an improvement of low-noise ERG in 11% and unchanged levels in 89% of patients in the HBO treatment group, while 62% of patients in the control group showed worsening of ERG and 38% remained unchanged with a significant difference between groups (*p* < 0.001) [6]. The other study compared HBO to vitamin A treatment and showed that HBO group had a slower decline in visual function, a higher percentage of visual field stabilization, and an improvement of low-noise ERG b-wave amplitude. However, in this study the dose regimen of the control group (vitamin A group) was not specified, the dropouts were not described, and the ERG instrument was changed after 3 years [5].

## 4. Discussion

Nineteen RCTs included in this review evaluated the effects of medical treatments on the progression of RP, including vitamins A and E, DHA, lutein, gangliosides' supplementation, HBO delivery, topical brimonidine tartrate treatment, intraocular CNTF release, oral nilvadipine, beta-carotene, and oral valproic acid. Among treatments evaluated in this systematic review, long term vitamin A supplementation showed a good short and long term safety profile but poor significant clinical result with the only significant effect being a slower rate of decline of ERG amplitude [28, 33, 35]. All the RCTs evaluating the efficacy and safety of DHA treatment compared to placebo or to vitamin A failed to demonstrate a beneficial effect of DHA supplementation, despite the good safety profile [26, 27, 29–31, 36]. The only beneficial effect was described by Berson et al. in a subgroup analysis, showing that patients with RP beginning vitamin A therapy plus DHA showed a better clinical outcome of the disease at 2 years [27]. Although a clear benefit on outcomes that would be clinically relevant for patients, such as visual acuity or visual field amplitude, has not been achieved, based on the results of all these studies, the evidence supports the supplementation of adults with early or middle stages of RP with 15000 IU of oral vitamin A palmitate every day, while supplementation with high dose vitamin E should be avoided [1, 8, 33, 35]. The encouraging results on the use of lutein supplementation in patients with RP obtained by Bahrami et al. were not confirmed by the subsequent RCT by Berson et al. that failed to demonstrate beneficial effects of lutein supplementation in terms of visual acuity and visual field [9, 10]. Other encouraging results come from small studies on 9-cis *β*-carotene-rich alga* Dunaliella bardawil* supplementation, oral valproic acid, and oral nilvadipine, which demonstrated to slow the decline of clinical outcomes in patients with RP [11–13]. However, these data should be confirmed by further larger, double-masked, controlled studies. The other medical treatments, including gangliosides' supplementation, topical brimonidine tartrate 0.2%, and intravitreal CNTF delivery, showed no beneficial effects on RP progression and clinical outcomes [14, 15, 32]. The latter even induced a worsening in visual field sensitivity when administered at higher doses [15].

Two RCTs by Vingolo et al. showed a significant improvement of low-noise ERG in patients with RP treated with HBO as compared to control untreated group [6]. In their later study, a slower progression in HBO group was also reported [5]. However, the conclusions of the authors are not definitive as they are not supported by data due to inappropriate statistical analysis and poor study design.

Nevertheless, we were unable to draw any conclusions at this time regarding the applicability or otherwise of the currently available medical interventions for RP because of the general paucity of evidence from the limited number of RCTs available and because of the differences between studies (e.g., different instruments and different outcome measures) that made it impossible to compare statistical data from different studies. Outcome assessment was mostly performed by visual acuity, visual field, or ERG evaluations; however, different instruments or different outcome measures were used for the same variable in different studies. For example, visual field was a common outcome assessed by Goldman perimetry or Humphrey visual field; however, while most of the studies using Goldman perimetry evaluated the visual field area, the studies using HFA evaluated the mean deviation or the total point score from different programs such as 10-2, 30-2, or 60-4. More standardized outcome measures to be used in RCT evaluation in RP patients should be assessed in the future, to make results reproducible and comparable. Owing to different formats of outcome reporting (e.g., mean rate of decline versus mean observed changes and continuous versus dichotomous data), different kinds of effect sizes had to be used, rendering both pooling and comparing of findings difficult.

Further RCTs with more standardized outcome measures and reporting are needed to definitely demonstrate the efficacy of the proposed therapeutic interventions and novel therapeutic agents aimed at improving visual function in patients with RP are highly sought after.

## Figures and Tables

**Table 1:** Study design and characteristics of randomized clinical trials included in the systematic review.

Randomized clinical trial	Masking	Treatments	Treatment duration	Time-point evaluation
Chatzinoff et al. 1968 [28]	Double-masked, controlled	100.000 U 11-cis vitamin A IM twice weekly versus 100.000 all-trans vitamin A IM twice weekly	3 years	Every six months for the duration of the study

Newsome et al. 1987 [32]	Double-masked, placebo-controlled	Gangliosides 40 mg/2 mL/day IM versus 0.9% sodium chloride solution/day IM injection	4 months	Baseline, 4 months.

Berson et al. 1993 [8]	Double-masked trial with 2 × 2 factorial design	Vitamin A 15,000 IU/day + vitamin E 3 UI/day (grA), vitamin A 75 IU/day + vitamin E 400 IU/day (grE), or vitamin A 15,000 IU/day + vitamin E 400 IU/day (grA + E) versus trace amounts of both vitamin A (75 IU/day) and vitamin E (3 UI/day) (trace group)	4 to 6 years	Each year for the duration of the study

Sibulesky et al. 1999 [33] Long term safety in the same population of Berson et al. 1993 [8]	Double-masked	Vitamin A 15,000 IU/day + vitamin E 3 UI/day (grA), vitamin A 75 IU/day + vitamin E 400 IU/day (grE), or vitamin A 15,000 IU/day + vitamin E 400 IU/day (grA + E) versus trace amounts of both vitamin A (75 IU/day) and vitamin E (3 UI/day) (trace group)	Up to 12 years	Baseline, 5 and 12 years

Vingolo et al. 1998 [6]	Unmasked study	Hyperbaric oxygen 90 min O_2_ daily 5 times a week for 1 month, 1 week a month for 11 months, and 1 week every 3 months for 2 years versus no hyperbaric oxygen	3 years	Each year for the duration of the study

Wheaton et al. 2003 [34] Long term safety in the same population of Hoffman et al. 2004 [30]	Double-masked, placebo-controlled	Oral DHA 400 mg/day supplementation versus placebo	4 years	Every 6 months for the duration of the study

Berson et al. 2004 [27]	Double-masked, controlled	Fatty acids with DHA 1200 mg/day + vitamin A 15000 UI/day versus fatty acids + vitamin A 15000 UI/day	4 years	Each year for the duration of the study

Berson et al. 2004 [26] Subgroup analysis of Berson et al. 2004 [27]: patients taking vitamin A prior to entry versus patients not taking vitamin A prior to entry	Double-masked, controlled	Fatty acids with DHA 1200 mg/day + vitamin A 15000 UI/day versus fatty acids + vitamin A 15000 UI/day	4 years	Each year for the duration of the study

Hoffman et al. 2004 [30]	Double-masked, placebo-controlled	Oral DHA 400 mg/day supplementation versus placebo	4 years	Each year for the duration of the study

Bahrami et al. 2006 [9]	Double-masked, crossover	Lutein capsule 10 mg/day for 12 weeks and then 30 mg/day for 12 weeks + multivitamin supplementation versus placebo capsule + multivitamin supplementation	6 months	Baseline, 6, 12, 18, and 24 weeks

Merin et al. 2008 [14]	Double masked, controlled	Brimonidine tartrate 0.2% eye drops BID versus artificial tears BID	2-3 years (mean: 29.3 months)	Every 6–8 months for the duration of the study

Vingolo et al. 2008 [5]	Unmasked, controlled	Hyperbaric oxygen 90 min O_2_ daily 5 days a week for 1 month, 5 consecutive days a month for 11 months, and 5 consecutive days every 3 months for 9 years versus vitamin A	10 years	Every 6 months for the duration of the study

Berson et al. 2010 [10]	Double masked, controlled	12 mg oral lutein + vitamin A 15000 UI/day versus placebo + vitamin A 15000 UI/day	4 years	Each year for the duration of the study

Nakazawa et al. 2011 [13]	Nonmasked	Oral nilvadipine 4 mg/day versus tocopherol nicotinate 300 mg/day or helenien 15 mg/day or no treatments	30–66 months	Every 6 months for the duration of the study

Rotenstreich et al. 2013 [11]	Double-masked, placebo-controlled, crossover	Four capsule/day of 9-cis *β*-carotene-rich alga *Dunaliella bardawil* (*β*-carotene, approximately 20 mg) versus 4-day capsule of placebo	3 months of treatment, 3 months of washout, 3 months of crossover	Baseline, after 3 months of treatment, after 3 months of washout, and after 3 months of crossover treatment

Birch et al. 2013 [15] Results of CNTF3 + CNTF4 studies	Masked, randomized, sham-controlled	High (20 ng/day) or low (5 ng/day) dose ciliary neurotrophic factor intraocular implant versus sham intraocular implant in the fellow eye	1 (CNTF3) or 2 years (CNTF4)	1 day, 1 week, 1, 3, 12, and 24 months, and follow-up up to 42 months

Hoffman et al. 2014 [29]	Double-masked, placebo-controlled	Multivitamin supplementation + DHA 30 mg/kg/day versus multivitamin supplementation + placebo capsules	4 years	Each year for the duration of the study

Hughbanks-Wheaton et al. 2014 [31] Same population of Hoffman et al. 2014 [29]	Double-masked, placebo-controlled	Multivitamin supplementation + DHA 30 mg/kg/day versus multivitamin supplementation + placebo capsules	4 years	Each year for the duration of the study

Kumar et al. 2014 [12]	Data evaluators, masked	Oral valproic acid 500 mg/day versus no treatment	1 year	Baseline, 3, 6, and 12 months

BID: Twice daily.

QD: Once a day.

QID: Four times daily.

**Table 2:** Characteristics of study population in the randomized clinical trials included in the systematic review.

Randomized clinical trial (country)	Population	Number of patients randomized per group (experimental/control)	Mean age of patients per group (years) (experimental/control)	Gender per group (experimental/control)
Chatzinoff et al. 1968 (USA) [28]	71 patients with RP	36/35	NA	NA

Newsome et al. 1987 (USA) [32]	32 patients with RP	17/15	43.7/39.8	10M, 7F/13M, 2F

Berson et al. 1993 [8] (USA)	601 patients with typical RP	Group A = 146, group E = 155, group A + E = 151, trace group = 149	Group A = 32.5, group E = 31.5, group A + E = 32.3, trace group = 32.2	373M, 228F

Sibulesky et al. 1999 [33] (USA)	121 patients with typical RP	Group A = 115, trace group = 106	NA	NA

Vingolo et al. 1998 [6] (ITA)	48 patients with typical RP	24/24	33.6/32.8	14M, 10F/16M, 8F

Wheaton et al. 2003 [34] and Hoffman et al. 2004 [30] (USA)	44 X-linked RP patients	23/21	14.9/18	23M/21M

Berson et al. 2004 (USA) [27]	208 patients with typical RP	105/103	37.8/36	50M, 55F/56M, 47F

Berson et al. 2004a^*∗*^ (USA) [26] Subgroup analysis of Berson et al. 2004 [27]	208 patients with typical RP	Vitamin A prior to entry: 75/68 No vitamin A prior to entry: 30/35	Vitamin A prior to entry: 38.1/36.8 No vitamin A prior to entry: 36.9/34.5	Vitamin A prior to entry: 37F, 38M/32F, 38M No vitamin A prior to entry: 18F, 12M/17F, 18M

Bahrami et al. 2006 [9] (USA)	34 patients with typical RP	16/18 crossover design	52.4/46.4	11F, 5M/10F, 8M

Merin et al. 2008 [14] (Israel)	17 patients with RP	17 patients randomized per eye	38	10M, 7F

Vingolo et al. 2008 [5] (ITA)	88 patients with RP	44/44	35/35.5	21M, 23F/21M, 23F

Berson et al. 2010 [10] (USA)	225 nonsmoker patients with typical RP	110/115	40/38	58M, 42F/52M, 63F

Nakazawa et al. 2011 [13] (Japan)	33 patients with RP	19/14	52/48	9M, 10F/7M, 7F

Rotenstreich et al. 2013 [11] (Israel)	29 patients with RP	16 *Dunaliella-*first group/18 placebo-first group	46.7	21M, 8F

Birch et al. 2013 [15] (USA)	65 patients with late-stage RP (CNTF3 study), and 68 patients with early-stage RP (CNTF4 study)	Low dose: 42 High dose: 91 Sham: 133 (fellow eye)	Low dose: 38 High dose: 41	Low dose: 24M, 18F High dose: 43M, 48F

Hoffman et al. 2014 [29] (USA) and Hughbanks-Wheaton et al. 2014 [31]	60 X-linked RP patients	27/33	16.1/14.9	27M/33M

Kumar et al. 2014 [12] (India)	30 patients with typical RP	15/15	30/31	10M, 5F/12M, 3F

RP: Retinitis pigmentosa.

CNTF: Ciliary neurotrophic factor.

M: Male.

F: Female.

^*∗*^Subgroup analysis of Berson 2004 study: patients taking vitamin A prior to entry versus patients not taking vitamin A prior to entry.

**Table 3:** Summary of results of the randomized clinical trials included in the systematic review.

Study	BCVA	Visual field	Contrast sensitivity	ERG	Dark adaptometry	Number of adverse events per group (experimental/control)
Chatzinoff et al. 1968 [28]	No differences	No differences	NA	NA	No differences	NA

Newsome et al. 1987 [32]	NA	No differences (Goldman perimetry)	NA	Not recordable in most patients	NA	3 minor AEs

Berson et al. 1993 [8] and Sibulesky et al. 1999 [33]	No differences (ETDRS)	No differences (Goldman perimetry)	NA	Slower rate of decline in vitamin A and vitamin A + E groups, faster decline in vitamin E group (30 Hz ERG)	NA	4 severe AEs (total), no differences between groups in minor and severe AE

Vingolo et al. 1998 [6]	NA	NA	NA	Improvement in maximal ERG amplitude recorded with low-noise method in HBO group	NA	NA

Berson et al. 2004 [27]	No differences (ETDRS)	No differences (Humphrey visual field analyzer)	NA	No differences (30 Hz ERG)	NA	1 severe AE in the placebo group

Berson et al. 2004a [26]	No differences (ETDRS)	Slower decline in no vitamin A prior to entry + DHA group (Humphrey visual field analyzer)	NA	Slower decline in no vitamin A prior to entry + DHA group (30 Hz ERG)	NA	1 severe AE in the placebo group

Hoffman et al. 2004 [30] and Wheaton et al. 2003 [34]	No differences (logMAR)	No differences (Humphrey visual field analyzer)	NA	No differences (light-adapted cone 31 Hz, rod, or maximal ERG amplitudes)	No differences	Minor AE in 4 patients in DHA and 6 in placebo groups. No severe AE

Bahrami et al. 2006 [9]	No differences (ETDRS)	Positive effect on preserving VF (Goldman perimetry)	No differences	NA	NA	Minor AE, 1/2

Merin et al. 2008 [14]	No differences (LogMar)	No differences (Goldman perimetry)	No differences	NA	NA	No severe AE Allergic reaction in 3

Vingolo et al. 2008 [5]	Slower decline in HBO group (Snellen chart)	Higher percentage of stabilization of Goldmann perimetry-target I4 and HFA 10-2 visual fields in the HBO group versus control group	NA	Statistically significant improvement of ERG b-wave amplitude recorded with low-noise method in HBO group versus controls	NA	NA

Berson et al. 2010 [10]	No differences (ETDRS)	No differences (Humphrey visual field analyzer)	NA	No differences		

Nakazawa et al. 2011 [13]	NA	Slower progression of central VF in treatment group (Humphrey visual field analyzer)	NA	NA	NA	No severe AE

Rotenstreich et al. 2013 [11]	No differences (ETDRS)	No differences in dark- and light-adapted VF (Goldmann perimetry)	NA	Significant increase of dark- and light-adapted ERG b-wave amplitude in treatment group versus placebo	NA	No AE

Birch et al. 2013 [15]	No differences (ETDRS)	No differences with low dose; significant worsening in high dose versus sham (Humphrey visual field analyzer)	NA	No differences (30 Hz flicker and single-flash ERG)	NA	Low dose: 5 ocular AEs High dose: 37 ocular AEs (26 mioses) No severe AE

Hoffman et al. 2014 [29]	NA	NA	NA	No differences (31 Hz flicker ERG)	NA	22/20 TEAEs No severe AE

Hughbanks-Wheaton et al. 2014 [31]	NA	NA	NA	NA	NA	22/20 TEAEs No severe AE 1 dropout due to gastrointestinal symptoms

Kumar et al. 2014 [12]	Significant improvement in treatment group	NA	NA	Significant improvement in treatment group	NA	3 gastrointestinal symptoms No severe AE

AE: Adverse event.

NA: Not assessed.

HBO: Hyperbaric oxygen therapy.

## References

[1] Hartong D. T., Berson E. L., Dryja T. P. (2006). Retinitis pigmentosa. *The Lancet*.

[2] Grover S., Fishman G. A., Anderson R. J. (1999). Visual acuity impairment in patients with retinitis pigmentosa at age 45 years or older. *Ophthalmology*.

[3] Berson E. L., Sandberg M. A., Rosner B., Birch D. G., Hanson A. H. (1985). Natural course of retinitis pigmentosa over a three-year interval. *American Journal of Ophthalmology*.

[4] Naash M. L., Peachey N. S., Li Z.-Y. (1996). Light-induced acceleration of photoreceptor degeneration in transgenic mice expressing mutant rhodopsin. *Investigative Ophthalmology and Visual Science*.

[5] Vingolo E. M., Rocco M., Grenga P. L., Salvatore S., Pelaia P. (2008). Slowing the degenerative process, long lasting effect of hyperbaric oxygen therapy in retinitis pigmentosa. *Graefe's Archive for Clinical and Experimental Ophthalmology*.

[6] Vingolo E. M., Pelaia P., Forte R., Rocco M., Giusti C., Rispoli E. (1998). Does hyperbaric oxygen (HBO) delivery rescue retinal photoreceptors in retinitis pigmentosa?. *Documenta Ophthalmologica*.

[7] Hamel C. (2006). Retinitis pigmentosa. *Orphanet Journal of Rare Diseases*.

[8] Berson E. L., Rosner B., Sandberg M. A. (1993). A randomized trial of vitamin A and vitamin E supplementation for retinitis pigmentosa. *Archives of Ophthalmology*.

[9] Bahrami H., Melia M., Dagnelie G. (2006). Lutein supplementation in retinitis pigmentosa: PC-based vision assessment in a randomized double-masked placebo-controlled clinical trial [NCT00029289]. *BMC Ophthalmology*.

[10] Berson E. L., Rosner B., Sandberg M. A. (2010). Clinical trial of lutein in patients with retinitis pigmentosa receiving vitamin A. *Archives of Ophthalmology*.

[11] Rotenstreich Y., Belkin M., Sadetzki S. (2013). Treatment with 9-cis beta-carotene-rich powder in patients with retinitis pigmentosa a randomized crossover trial. *JAMA Ophthalmology*.

[12] Kumar A., Midha N., Gogia V., Gupta S., Sehra S., Chohan A. (2014). Efficacy of oral valproic acid in patients with retinitis pigmentosa. *Journal of Ocular Pharmacology and Therapeutics*.

[13] Nakazawa M., Ohguro H., Takeuchi K., Miyagawa Y., Ito T., Metoki T. (2011). Effect of nilvadipine on central visual field in retinitis pigmentosa: a 30-month clinical trial. *Ophthalmologica*.

[14] Merin S., Obolensky A., Farber M. D., Chowers I. (2008). A pilot study of topical treatment with an alpha2-agonist in patients with retinal dystrophies. *Journal of Ocular Pharmacology and Therapeutics*.

[15] Birch D. G., Weleber R. G., Duncan J. L., Jaffe G. J., Tao W. (2013). Randomized trial of ciliary neurotrophic factor delivered by encapsulated cell intraocular implants for retinitis pigmentosa. *American Journal of Ophthalmology*.

[16] Akiyama M., Ikeda Y., Yoshida N. (2014). Therapeutic efficacy of topical unoprostone isopropyl in retinitis pigmentosa. *Acta Ophthalmologica*.

[17] Berson E. L., Rosner B., Sandberg M. A., Weigel-DiFranco C., Willett W. C. (2012). *ω*-3 Intake and visual acuity in patients with retinitis pigmentosa receiving vitamin A. *Archives of Ophthalmology*.

[18] Yamamoto S., Sugawara T., Murakami A. (2012). Topical isopropyl unoprostone for retinitis pigmentosa: microperimetric results of the phase 2 clinical study. *Ophthalmology and Therapy*.

[19] Aleman T. S., Duncan J. L., Bieber M. L. (2001). Macular pigment and lutein supplementation in retinitis pigmentosa and usher syndrome. *Investigative Ophthalmology and Visual Science*.

[20] Harding C. O., Gillingham M. B., van Calcar S. C., Wolff J. A., Verhoeve J. N., Mills M. D. (1999). Docosahexaenoic acid and retinal function in children with long-chain 3-hydroxyacyl-CoA dehydrogenase deficiency. *Journal of Inherited Metabolic Disease*.

[21] Khavinson V., Razumovsky M., Trofimova S., Grigorian R., Razumovskaya A. (2002). Pineal-regulating tetrapeptide epitalon improves eye retina condition in retinitis pigmentosa. *Neuroendocrinology Letters*.

[22] Pasantes-Morales H., Quiroz H., Quesada O. (2002). Treatment with taurine, diltiazem, and vitamin E retards the progressive visual field reduction in retinitis pigmentosa: a 3-year follow-up study. *Metabolic Brain Disease*.

[23] Rotenstreich Y., Harats D., Shaish A., Pras E., Belkin M. (2010). Treatment of a retinal dystrophy, fundus albipunctatus, with oral 9-cis-*β*-carotene. *The British Journal of Ophthalmology*.

[24] Saltzman S. L., Haig C. (1950). Treatment of retinitis pigmentosa with cod liver oil injection and placental implantation. *Archives of Ophthalmology*.

[25] Nakazawa M., Suzuki Y., Ito T., Metoki T., Kudo T., Ohguro H. (2013). Long-term effects of nilvadipine against progression of the central visual field defect in retinitis pigmentosa: an extended study. *BioMed Research International*.

[26] Berson E. L., Rosner B., Sandberg M. A. (2004). Further evaluation of docosahexaenoic acid in patients with retinitis pigmentosa receiving vitamin A treatment: subgroup analyses. *Archives of Ophthalmology*.

[27] Berson E. L., Rosner B., Sandberg M. A. (2004). Clinical trial of docosahexaenoic acid in patients with retinitis pigmentosa receiving vitamin A treatment. *Archives of Ophthalmology*.

[28] Chatzinoff A., Nelson E., Stahl N., Clahane A. (1968). Eleven-CIS vitamin A in the treatment of retinitis pigmentosa. A negative study. *Archives of Ophthalmology*.

[29] Hoffman D. R., Hughbanks-Wheaton D. K., Pearson N. S. (2014). Four-year placebo-controlled trial of docosahexaenoic acid in X-linked retinitis pigmentosa (DHAX Trial): a randomized clinical trial. *JAMA Ophthalmology*.

[30] Hoffman D. R., Locke K. G., Wheaton D. H., Fish G. E., Spencer R., Birch D. G. (2004). A randomized, placebo-controlled clinical trial of docosahexaenoic acid supplementation for X-linked retinitis pigmentosa. *The American Journal of Ophthalmology*.

[31] Hughbanks-Wheaton D. K., Birch D. G., Fish G. E. (2014). Safety assessment of docosahexaenoic acid in X-linked retinitis pigmentosa: the 4-year DHAX trial. *Investigative Ophthalmology and Visual Science*.

[32] Newsome D. A., Dorsey F. C., May J. G., Bergsma D. R., Bazan N. G. (1987). Ganglioside administration in retinitis pigmentosa. *Journal of Ocular Pharmacology*.

[33] Sibulesky L., Hayes K. C., Pronczuk A., Weigel-DiFranco C., Rosner B., Berson E. L. (1999). Safety of <7500 RE (<25000 IU) vitamin A daily in adults with retinitis pigmentosa. *The American Journal of Clinical Nutrition*.

[34] Wheaton D. H., Hoffman D. R., Locke K. G., Watkins R. B., Birch D. G. (2003). Biological safety assessment of docosahexaenoic acid supplementation in a randomized clinical trial for X-linked retinitis pigmentosa. *Archives of Ophthalmology*.

[35] Berson E. L., Rosner B., Sandberg M. A. (1993). Vitamin A supplementation for retinitis pigmentosa. *Archives of Ophthalmology*.

[36] Hoffman D. R., DeMar J. C., Heird W. C., Birch D. G., Anderson R. E. (2001). Impaired synthesis of DHA in patients with X-linked retinitis pigmentosa. *Journal of Lipid Research*.

